# Impact of a United Kingdom-wide campaign to tackle antimicrobial resistance on self-reported knowledge and behaviour change

**DOI:** 10.1186/s12889-016-3057-2

**Published:** 2016-05-12

**Authors:** Katerina Chaintarli, Suzanne M. Ingle, Alex Bhattacharya, Diane Ashiru-Oredope, Isabel Oliver, Maya Gobin

**Affiliations:** Field Epidemiology Service, Public Health England, Bristol, UK; Health Protection Research Unit in Evaluation of Interventions, School of Social and Community Medicine, University of Bristol, Bristol, UK; Antimicrobial Resistance Programme, Public Health England, London, UK

**Keywords:** Public health, Campaign evaluation, Behavioral medicine, Pledge scheme, Behaviour change, Health promotion, Health campaign, Implementation intentions, Antibiotic resistance

## Abstract

**Background:**

As part of the 2014 European Antibiotic Awareness Day plans, a new campaign called Antibiotic Guardian (AG) was launched in the United Kingdom, including an online pledge system to increase commitment from healthcare professionals and members of the public to reduce antimicrobial resistance (AMR). The aim of this evaluation was to determine the impact of the campaign on self-reported knowledge and behaviour around AMR.

**Methods:**

An online survey was sent to 9016 Antibiotic Guardians (AGs) to assess changes in self-reported knowledge and behaviour (outcomes) following the campaign. Logistic regression models, adjusted for variables including age, sex and pledge group (pledging as member of public or as healthcare professional), were used to estimate associations between outcomes and AG characteristics.

**Results:**

2478 AGs responded to the survey (27.5 % response rate) of whom 1696 (68.4 %) pledged as healthcare professionals and 782 (31.6 %) as members of public (similar proportions to the total number of AGs). 96.3 % of all AGs who responded had prior knowledge of AMR. 73.5 % of participants were female and participants were most commonly between 45 and 54 years old.

Two thirds (63.4 %) of participants reported always acting according to their pledge. Members of the public were more likely to act in line with their pledge than professionals (Odds Ratio (OR) =3.60, 95 % Confidence Interval (CI): 2.88-4.51). Approximately half of participants (44.5 %) (both healthcare professionals and members of public) reported that they acquired more knowledge about AMR post-campaign. People that were confused about AMR prior to the campaign acquired more knowledge after the campaign (OR = 3.10, 95 % CI: 1.36-7.09). More participants reported a sense of personal responsibility towards tackling AMR post-campaign, increasing from 58.3 % of participants pre-campaign to 70.5 % post-campaign.

**Conclusion:**

This study demonstrated that the campaign increased commitment to tackling AMR in both healthcare professional and member of the public, increased self-reported knowledge and changed self-reported behaviour particularly among people with prior AMR awareness. Online pledge schemes can be an effective and inexpensive way to engage people with the problem of AMR especially among those with prior awareness of the topic.

**Electronic supplementary material:**

The online version of this article (doi:10.1186/s12889-016-3057-2) contains supplementary material, which is available to authorized users.

## Background

Antimicrobial resistance (AMR) is a major public health problem and is partly associated with high levels of antimicrobial use [1–3]. Prescribing practices in humans and animals have led to the overuse and misuse of antimicrobials and the development of AMR [4–7]. AMR remains an important issue for the United Kingdom (UK) despite a reduction in the total consumption of antibiotics in recent years [8, 9]. The World Health Organisation (WHO) Global Strategy for Containment of Antimicrobial Resistance provides a framework for interventions to slow the emergence and reduce the spread of AMR [10, 11]. One aim of the strategy includes actions to educate members of the public and healthcare professionals about AMR [11]. Similarly in the UK, the 5 year UK AMR strategy (2013) includes improving professional education, training and public engagement as one of its seven key areas [12].

Numerous educational campaigns have been implemented worldwide to help tackle AMR with differing levels of effectiveness [6, 13–21]. The “European Antibiotic Awareness Day” (EAAD) was initiated by the European Centre for Disease Prevention and Control to raise awareness towards antibiotic use [22, 23]. The UK “Antibiotic Guardian Campaign” was launched in September 2014 to increase public and healthcare professionals engagement and change behaviour towards the rising threat of AMR [24]. It was also developed as a way to have, for the first time an ‘always on’ (available all year round) campaign for AMR. The campaign comprised an online pledge system (http://antibioticguardian.com/) and was available to everyone with access to the internet. People could select a single pledge from a list tailored for either members of the public or health care professionals (the third group “student or educator” was added in October 2015 - after the evaluation of the campaign had started) (Additional file 1). Online pledges were one of the recommended interventions from a recent behavioural analysis that identified key behaviours and drivers for antibiotic use in both the public and healthcare professionals [25, 26]. The Antibiotic Guardian pledges were subsequently designed to overcome the intention – behaviour gap through the formation of implementation intentions (or if – then plans) [27]. Implementation intention is a method of deciding in advance when, where and how a person will act on particular action to reach a particular goal or objective using If-then approach - If **X** happens, then I will do **Y**. For example, one of the Antibiotic Guardian pledges for members of the public states: ‘For infections that our bodies are good at fighting off on their own, like coughs, colds, sore throats and flu, I pledge to talk to my pharmacist about how to treat the symptoms first rather than going to the GP’ and for a general practitioner (GP) one of the pledges available to select from is ‘The next time I intend to prescribe antibiotics for a self-limiting infection to a patient with high expectations of antibiotic treatment, I will use a delayed/backup prescription’. Using implementation intentions have been shown through several meta-analyses to support individuals as well as groups in their intention-behaviour gaps [28, 29].

The initial concept for the website and logo was developed jointly with the British Society for Antimicrobial Chemotherapy, who also kindly provided the initial funding for the website. The campaign website was launched by Public Health England (PHE) alongside the release of digital resources such as posters, leaflets and social media graphics developed at no additional cost by PHE which were shared openly for use [30]. The AG website included a video produced by PHE which educated on the threat of antimicrobial resistance and linked to resources which described the importance of AMR [23]. All materials were made available via the English EAAD website and EAAD resources toolkit for health care professionals [23]. PHE wrote letters to leaders in primary and secondary care and professional organisations with the purpose of forming a network through which messages and materials could be promoted and disseminated [31]. Further promotion for the campaign by PHE took place at professional-facing conferences through donated exhibition space. The 182 organisations that registered participation for EAAD by 30th November were also invited to promote the AG campaign to staff and service users [23].

The main objective of that campaign was that by 30th November 2014 at least 10,000 healthcare professionals and members of the public would have committed to at least one pledge for prudent use of antimicrobials. The objective was met on the 19th November and by the end of November there were 11,833 Antibiotic Guardians.

The aim of this evaluation was to determine whether the AG campaign increased engagement and improved AMR knowledge and behaviour.

## Methods

An online questionnaire was developed to determine if the campaign resulted in changes in self-reported knowledge/awareness and self-reported behaviour amongst Antibiotic Guardians (AGs) (Additional file 2). AGs who previously consented to follow up were sent the questionnaire via e-mail on 3rd February 2015, five months after the launch of the campaign. The questionnaire was available for completion for one month; a reminder was sent via e-mail a week after the launch of the survey. Before launching the survey, the questionnaire was reviewed by a wide range of healthcare professionals and members of the public who were members of the EAAD planning group.

Demographics of the pledge group (pledging as a health professional or a member of the public), age, sex, use of social media and whether working in a health related profession were collected (Questions 1–3 & 21–27). Closed multi-choice questions were used to ascertain the participant’s motivation for becoming an Antibiotic Guardian and the reason for choosing their specific pledge (Questions 4–6). Participants were asked to select the options that best described their behaviour in relation to the pledge (‘Pledge Behaviour’) and their general knowledge/awareness of AMR before and after becoming an Antibiotic Guardian (pre and post-campaign) (Questions 7–14).

The perceived effectiveness of the promotion of the campaign and quality of the promotional material were ascertained through a series of multiple choice questions (Questions 15–20). The questionnaire logic was designed such that some questions were skipped depending on whether the participant had pledged as a member of the public or as a healthcare professional.

The two primary outcomes “change in self-reported behaviour” and “change in knowledge” were assessed as the difference in reported frequency of pledge behaviour pre and post campaign and whether participants stated a positive acquisition of knowledge post-campaign respectively.

### Statistical analysis

To assess bias in our sample, we compared the response rates of members of public and health care professionals among those who completed the survey with the total population that signed up as an AG.

To assess the outcome ‘change in behaviour’ after the campaign, we used an ordinal logistic regression model [32] to estimate associations with the ordered categorical outcome “acting in line with their pledge” (Question 7). The model was adjusted for variables describing whether participants acted according to their pledge before the campaign and whether they remembered their pledge (Questions 4 & 14). Multivariable analysis was conducted taking into account pledge group, age and sex as possible confounders.

To assess the outcome ‘knowledge of AMR after the campaign’ we used a logistic regression model to estimate associations with the outcome (Question 12). We looked for associations with knowledge before the campaign and whether people were confused about what AMR is before the campaign (Questions 10 & 11). Multivariable analysis was conducted taking into account pledge group, age and sex as possible confounders.

For all categorical variables, the group with the largest number of observations was used as the reference group for each variable.

### Missing values

After assessing the amount of missing data from our models, we decided to perform some sensitivity analysis to assess whether our complete-case study could be biased. We assumed that data were missing at random, although this cannot be tested [33]. A multiple imputation model was developed using chained equations and the ice command in Stata. The model included all the covariates and outcomes of interest (as listed in Table 1) as well as a comprehensive (but not exhaustive) list of other variables collected. 20 imputed datasets were created and were analysed following Rubin’s rules.

**Table 1:** Baseline characteristics of survey participants, Number of observations (*N*) = 2478

Variable	*N* (%)
Pledge group	
Healthcare Professionals	1696 (68.4 %)
Members of Public	782 (31.6 %)
Missing	0 (0 %)
Age	
<35 years old	596 (24.1 %)
35-64 years old	1548 (62.5 %)
≥65 years old	114 (4.6 %)
Prefer not to say	10 (0.4 %)
Missing	210 (8.5 %)
Sex	
Female	1657 (66.9 %)
Male	598 (24.1 %)
Prefer not to say	15 (0.6 %)
Missing	208 (8.4 %)
Do you remember your pledge?	
Yes	1087 (43.9 %)
Somewhat	1286 (51.9 %)
No	105 (4.2 %)
Missing	0 (0 %)
Act in line with pledge before campaign	
Strongly agree	761 (30.7 %)
Agree	1021 (41.2 %)
Tend to Agree	505 (20.4 %)
Tend to Disagree	101 (4.1 %)
Disagree	6 (0.2 %)
Strongly Disagree	4 (0.2 %)
Missing	80 (3.2 %)
Confusion on what AMR is before the campaign?	
Yes	34 (1.4 %)
No	2399 (96.8 %)
Missing	45 (1.8 %)
Prior knowledge on AMR?	
Yes	2386 (96.3 %)
No	44 (1.8 %)
Missing	48 (1.9 %)
Outcomes	
Act in line with pledge after campaign	
Always	1571 (63.4 %)
Most of the time	709 (28.6 %)
Some of the time	96 (3.9 %)
Occasionally	64 (2.6 %)
Never	37 (1.5 %)
Missing	1 (0.0 %)
Acquired more knowledge of AMR after campaign	
No	1316 (53.1 %)
Yes	1103 (44.5 %)
Missing	59 (2.4 %)

Data were analysed using Stata 13.1 [34].

## Results

The questionnaire was completed by 2478 AGs; 27.5 % (2478/9016) of those that consented for follow up and received the questionnaire. Response rates in each pledge group were similar (20.7 % for healthcare professionals and 21.5 % for members of the public). Table 1 shows the baseline characteristics of the 2478 AGs. Two thirds of participants pledged as healthcare professionals (68.4 %). Of those AGs with valid data, the majority were females (73.5 %) and most participants were between 45 and 54 years old.

Of the 782 people who pledged as members of public, 134 (17.1 %) were healthcare professionals and 208 (26.6 %) were connected to the healthcare system (e.g., working in a healthcare organisation or health related professional body). Characteristics of the survey participants were similar to those of the total population of Antibiotic Guardians (Graph 1 shows job type for healthcare professionals and certain job types/family status for members of the public).

The majority of public participants (64.2 %) became Antibiotic Guardians because they knew the importance of antimicrobial resistance. For healthcare professionals the reason most commonly stated was professional experience of AMR (32.5 %). For both healthcare professionals and members of public, the majority of participants were able to remember their pledge either in part (51.9 %) or completely (43.9 %). Also, for both groups in total, most participants chose the specific pledge because it was the one they could commit to (57.5 %), or felt was most important (31.7 %). Almost all participants of both groups (91.3 %) felt that the AG campaign would contribute to the prevention of antibiotic resistance and they were part of a wider community of people working to keep antibiotics active (88.9 %).

### Change in self-reported behaviour

Self-reported action in line with their pledge increased from 30.7 % pre-campaign to 63.4 % post-campaign. Pledge group, pre-campaign reported pledge behaviour and remembering the pledge were associated with post-campaign reported pledge behaviour in the multivariable analysis (Table 2). Members of the public were more likely to report positive post-campaign pledge behaviour (Adjusted Odds Ratio (aOR) = 3.60, 95 % Confidence interval (CI): 2.88-4.51). Participants without positive pre-campaign pledge behaviour were less likely to have positive post-campaign pledge behaviour (aOR = 0.23, 95 % CI: 0.16-0.34). AGs who remembered the pledge were more likely to have positive post-campaign behaviour (aOR = 1.96, 95 % CI: 1.63-2.36). Age and sex were not associated with post-campaign pledge behaviour.

**Table 2:** Crude and Adjusted Odds Ratios from ordinal logistic regression model for associations with Antibiotic Guardians acting according to their pledge, *N* = 2242

Covariate	*N*	Crude OR (95 % CI)	aOR (95 % CI)
Act according to pledge before becoming AG			
Yes	2141	1.0 (ref)	1.0 (ref)
No	101	0.29 (0.20 – 0.43)	0.23 (0.16 – 0.34)
Remember the pledge			
Completely	994	1.63 (1.37 – 1.95)	1.96 (1.63 – 2.36)
Somewhat	1163	1.0 (ref)	1.0 (ref)
No	85	0.28 (0.18 – 0.46)	0.24 (0.15 – 0.38)
Pledge group			
Members of Public	708	3.09 (2.50 – 3.82)	3.60 (2.88 – 4.51)
Healthcare Professionals	1534	1.0 (ref)	1.0 (ref)
Age			
<35 years old	592	0.94 (0.77 – 1.14)	0.86 (0.70 – 1.05)
35-64 years old	1530	1.0 (ref)	1.0 (ref)
≥65 years old	110	1.63 (1.04 – 2.54)	1.11 (0.68 – 1.79)
Prefer not to say	10	0.36 (0.11,1.16)	0.36 (0.10,1.24)
Sex			
Female	1641	1.0 (ref)	1.0 (ref)
Male	591	0.99 (0.82 – 1.20)	1.09 (0.89 – 1.33)
Prefer not to say	10	2.03 (0.43-9.66)	3.92 (0.61-25.11)

*N* number of observations, *OR* odds ratio, *aOR* adjusted OR for all variables listed in the table

Members of the public stated that lack of opportunity was the most common reason (60 %) for the absence of positive post-campaign pledge behaviour. For health professionals, the expectations of others (mainly patients) was the most common reason stated for not fulfilling their pledge (40.4 %).

### Knowledge/awareness of AMR

Approximately half of participants (44.5 %) (both healthcare professionals and members of public) reported that they acquired more knowledge about AMR post-campaign. More participants reported a sense of personal responsibility towards tackling AMR post-campaign, increasing from 58.3 % of participants pre-campaign to 70.5 % post-campaign. Of the participants who had not acquired more knowledge post-campaign, the majority (99.3 %) reported having good knowledge/awareness of AMR pre-campaign.

Table 3 shows that participants were more likely to have more knowledge about AMR post-campaign if they did not have pre-campaign knowledge (aOR = 4.21, 95 % CI: 2.04 – 8.67) or had some knowledge but were confused about AMR (aOR =3.10, 95 % CI: 1.36 – 7.09). Members of the public were less likely to have acquired more knowledge post-campaign than healthcare professionals (aOR = 0.79, 95 % CI: 0.66 – 0.96). Age and sex were not associated with the acquisition of more knowledge post-campaign.

**Table 3:** Crude and Adjusted Odd Ratios from logistic regression model for associations with knowledge on antimicrobial resistance after the Antibiotic Guardian campaign, *N* = 2224

Covariate	N	Crude OR (95 % CI)	aOR (95 % CI)
Confused about AMR before the campaign			
Yes	29	3.21 (1.41 – 7.27)	3.10 (1.36 – 7.09)
No	2195	1.0 (ref)	1.0 (ref)
Prior knowledge on AMR			
Yes	2182	1.0 (ref)	1.0 (ref)
No	42	3.95 (1.93 – 8.07)	4.21 (2.04 – 8.67)
Pledge group			
Members of Public	700	0.83 (0.70 – 1.00)	0.79 (0.66 – 0.96)
Healthcare Professionals	1524	1.0 (ref)	1.0 (ref)
Age			
<35 years old	585	1.13 (0.93 – 1.36)	1.12 (0.93 – 1.36)
35-64 years old	1521	1.0 (ref)	1.0 (ref)
≥65 years old	108	0.78 (0.52 – 1.16)	0.88 (0.58 – 1.33)
Prefer not to say	10	0.82 (0.23-2.91)	1.13 (0.29-4.41)
Sex			
Female	1629	1.0 (ref)	1.0 (ref)
Male	585	0.92 (0.76 – 1.11)	0.89 (0.73 – 1.08)
Prefer not to say	10	0.29 (0.06-1.38)	0.29 (0.06-1.43)

*N* number of observations, *OR* odds ratio, *aOR* adjusted OR for all variables listed in the table

### Promotion

Over half of the participants for both healthcare professionals and members of public (61.7 %) thought the AG campaign was well promoted in terms of motivating them to take the pledge although more than half of them (60.6 %) had not seen some of the promotional materials such as the quiz or the crosswords. A greater proportion of members of the public had not seen the different promotional materials (43.2 %) as compared with healthcare professionals (33.9 %), possibly because the campaign was mainly promoted through healthcare settings. The majority of participants said they were or would be willing to actively promote the campaign to friends, family and colleagues.

Overall participants stated that information from a combination of sources influenced their decision to become AGs. Table 4 shows that age was associated with the type of information source with biggest impact (Chi2 tests P <0.001). Under 18 year olds were most influenced by social media and the website whereas older participants were most influenced by colleagues or a combination of resources.

**Table 4:** Sources that made the biggest impact on becoming an Antibiotic Guardian in relation to age and use of social media

	Sources that made the biggest impact on becoming an Antibiotic Guardian (%)							
	Printed materials	YouTube	Social media	Website	Colleagues	Friends	Combination	None
Age								
12-17 years old	0.0	0.0	28.6	28.6	0.0	28.6	14.3	0.0
18-24 years old	4.6	2.0	12.4	17.7	22.2	5.2	30.7	5.2
25-34 years old	4.6	2.3	16.2	15.2	26.6	2.8	21.3	11.1
35-44 years old	3.8	1.4	17.1	15.1	21.7	1.4	28.2	11.3
45-54 years old	4.9	1.3	8.6	18.7	20.2	1.6	33.9	10.8
55-64 years old	8.4	0.3	7.1	17.9	17.1	1.9	36.3	11.1
65-74 years old	13.9	1.0	8.9	17.8	8.9	5.0	24.8	19.8
75 years or older	15.4	0.0	0.0	7.7	7.7	7.7	46.2	15.4
Total	5.6	1.4	12.2	16.9	20.8	2.4	29.8	11.1
Social Media User								
Yes	4.5	1.5	15.5	15.9	19.8	2.6	29.9	10.2
No	9.3	1.0	0.2	19.9	23.6	1.8	29.5	14.8
Total	5.6	1.4	12.1	16.8	20.7	2.4	29.9	11.3

#### Missing data analysis

Looking at the pattern of missing data, 89 % of the records have complete data on the variables of interest. The analysis was rerun using the imputed data and the results were compared to the results of the original dataset and were found to be similar.

## Discussion

The AG campaign is the first published example of the use of an online pledge system to improve AMR related knowledge and behaviour amongst healthcare professionals and the general public. Our evaluation found that the AG campaign contributed to an overall increase in the personal commitment of healthcare professionals and members of the public to tackle AMR as well as improved self-reported AMR related behaviour and knowledge in both healthcare professionals and the general public who participated in the campaign.

There was a difference in the level of reported change in behaviour observed between healthcare professionals and members of the public. Healthcare professionals were less likely to act according to their pledge than members of the public. Literature shows that healthcare professionals’ perceptions about patient and professional expectations continue to influence their AMR related behaviour [35–38]. The last is also reflected by the results of our survey where the expectations of others was the most common reason stated among healthcare professionals for not fulfilling their pledge. A recommendation for next campaigns would be to work with the relevant groups to ensure that the pledges are achievable and realistic.

### Strengths and weaknesses of the campaign

The evaluation was conducted five months after the AG campaign and only provides an assessment of the short term impact of the campaign on self-reported behaviour. The campaign did not include reminding the AGs of their pledge and whilst a high proportion could remember their pledge in part less than half of the participants could remember their pledge completely. The launch of the AG campaign in the UK deliberately coincided with the 2014 European Antibiotic Awareness Day (EAAD) to move from awareness raising to increasing engagement and commitment and to provide an ‘always on’ AMR campaign that was available all year round. As part of the EAAD, European countries are provided with resources to inform people about AMR and on the prudent use of antibiotics although these resources were published later than the AG campaign.

We found the campaign contributed to increasing the personal commitment of healthcare professionals and members of the public as well as improvement in self-reported knowledge and awareness of AMR and was particularly effective for people who were previously confused about the subject. In general participants felt that the AG campaign was well promoted, however, our evaluation suggests that the campaign was less successful in engaging people without previous professional or personal experience of antimicrobial resistance. This likely to be due to the routes of promoting the campaign which relied on health and professional organisations rather than a public facing campaign using, for example, mass media.

The AGs who responded to the survey were similar in terms of pledge group to the total AG population. The current evaluation indicated that a pledge scheme can be an inexpensive and effective way of promoting knowledge, raising awareness and changing self-reporting behaviour on important public health issues especially among people with prior awareness of the topic.

However, certain caveats should be considered when reviewing the results. The majority of participants were female and aged between 44 and 54 years old, two demographic groups shown to be associated with better AMR related knowledge and behaviour [39, 40]. Details of the age and sex distribution of AGs were not available and comparison with the survey participants was not possible and non-responder bias cannot be excluded. However, it is known that there is a greater proportion of women between 35 and 54 years old among healthcare professionals [41, 42]. It is important for the next campaign to identify ways of engaging men and people of older age groups more effectively.

The evaluation was based on self-reported changes in behaviour and knowledge. It is possible that acquiescence bias was present as participants would have been aware of the desirability of a positive change in behaviour and knowledge around AMR. Recall bias may be considered but the majority of participants were able to remember their pledge either in part or completely five months after taking their pledge.

The online questionnaire used for our evaluation was a convenient and inexpensive way to quickly communicate with a large number of AGs but risks a lower response rate [43]. In addition, people who may have limited access to internet such as those from older age groups may be under-represented in such surveys. Other approaches may be needed to have an impact on those with limited internet access.

Overall, the survey was well completed; however, there was some missing information in questions pertaining to the promotion of the AG campaign and demographic characteristics of the participants. We have no reason to believe that these data are not missing at random (i.e., that men are less likely to answer the gender question) although this cannot be formally tested. Therefore, we imputed the missing data to reduce the chance of bias and improve power [33]. Analyses on the imputed data showed similar results to those reported here. In the paper, only the analysis of original data is reported.

Our evaluation provides the baseline characteristics of the participants and a high level assessment of the promotion of the campaign and impact on behaviour and knowledge. Qualitative methods will be applied to further investigate the results from the quantitative analysis.

### Context

The discovery of new antibiotics has proved challenging [3] and a variety of different campaigns to help tackle AMR have previously been developed; however a recent summary of the campaigns in the UK highlight that although the campaigns have increased awareness, there has been limited impact on increasing knowledge and changing behaviour [13]. Multifaceted educational programmes and antimicrobial stewardship interventions targeting healthcare professionals are associated with improved antimicrobial use [44, 45]. Mass media interventions as part of a multimodal campaign can also play an important role in educating healthcare professionals and especially members of public about AMR [13, 21, 45–48]. The AG campaign is the first published example of the use of a pledge system to engage, educate and encourage others towards positive behaviour change with regards to antibiotic prescribing, expectation and use [24].

Pledges have been used in a number of different contexts to effect behaviour change at an individual and organisational level [49–53]. Pledges are employed primarily for four purposes: increase awareness, change behaviour, engage others and collect data on individuals [54]. Only a limited number of pledge schemes have been evaluated and the evidence of effectiveness of pledge schemes is inconsistent [54].

Factors that contribute to successful behaviour change include direct personal contact with pledgees and prior awareness of/engagement with the topic and reflect the findings from our evaluation [54]. Although the majority of participants could remember the general meaning of their chosen pledge, over half could not remember in full their pledge at five months. The last indicates the need for regular communication with pledgees via e-mails or newsletters to remind them of their pledge and provide information on the topic. We also found that the campaign was primarily adopted by people with prior awareness of the topic which suggests that similar pledge-based campaigns may not be the most appropriate intervention to engage people with no prior awareness. Identifying alternative promotional methods to reach members of the public would be an important next step for the campaign.

Any new or ongoing pledge campaigns should adopt the lessons learnt from our evaluation and the techniques that have been successfully implemented in other similar campaigns [55, 56]. These include allowing pledgees to create personalised pledges and getting individuals to make pledges in public [57, 58]. Finally our evaluation was developed after the campaign was delivered and we recommend that evaluations be built into the design of future campaigns to effectively capture pre and post-campaign effects on behaviour and knowledge [13]. The next goal for the AG campaign 2015 is to have 100,000 Antibiotic Guardians by the end of March 2016 and subsequently to assess the impact of the campaign on outcomes such as reduced antibiotic use or prescribing.

## Conclusions

In conclusion, although there are limitations, we found that the Antibiotic Guardian campaign was effective in demonstrating engagement from healthcare professionals and members of the public to tackle antimicrobial resistance and also highlighted an increase in self-reported commitment of healthcare professionals and members of the public in their personal actions to tackle antimicrobial resistance. It was also effective for achieving positive changes in self-reported knowledge and behaviour amongst people with prior awareness of the topic. This approach could be developed further to engage with other groups including those without prior knowledge of AMR.

### Ethics

The project was a service evaluation of intervention and no ethics approval was required.

## Availability of supporting data

Data can be requested through Public Health England.

## Additional files

Additional file 1: Table S1. Antibiotic Guardian pledges by target audiences. (PDF 166 kb) Additional file 2: Online questionnaire used for the evaluation of the Antibiotic Guardian Campaign. (PDF 423 kb)

## Abbreviations

AG: antibiotic guardian
AMR: antimicrobial resistance
aOR: adjusted odds ratio
CI: confidence interval
EAAD: European Antibiotic Awareness Day
OR: odds ratio
UK: United Kingdom
WHO: World Health Organisation
