# Supplementary material for: Impact of a United Kingdom-wide campaign to tackle antimicrobial resistance on self-reported knowledge and behaviour change
Source: BMC Public Health. 2016 May 12;16:393. doi: 10.1186/s12889-016-3057-2 (PMC4866421; doi:10.1186/s12889-016-3057-2)
Supplement: Additional file 1: Table S1. — Antibiotic Guardian pledges by target audiences. (PDF 166 kb) [file 12889_2016_3057_MOESM1_ESM.pdf]

Supplementary Table. Antibiotic Guardian pledges by target audiences

Group	Pledge
Antimicrobial Pharmacist / Infection Prevention and Control Specialist	I will encourage and champion members of my organisation to become Antibiotic Guardians
	I will lead promotional activities for EAAD within my organisation
	I will create a written action plan for the implementation of SSTF or TARGET within my organisation
Dentists	I will encourage clients/patients and colleagues to become Antibiotic Guardians
	When I see a patient with dental pain, I will discuss methods of controlling symptoms rather than prescribing antibiotics as a first course of action
	I will consider drainage for dental infections before issuing antibiotics
Executives / Management / Government / Commissioners/ Public Health	I will champion promotional activity for EAAD within my local area or organisation
	I pledge to incorporate Antimicrobial Stewardship and Resistance as a quality measure within my commissioning pathways (including Out of Hours)
	I will ensure that the executive team and board are regularly informed about Antimicrobial Stewardship and AMR in my Trust
	I will visit my local hospital, community pharmacy or general practice surgery to show support for European Antibiotics Awareness Day during EAAD week (18-22 November)
	I will encourage implementation of Start Smart then Focus guidance within my organisation
Non-Medical Prescribers	When I write an antimicrobial prescription I will make sure it’s in line with local guidelines
	I will undertake one of the recommended CPD activities recommended on the EAAD resources page during EAAD week
	The next time I want to prescribe an antibiotic for a self-limiting infection for a patient with high expectations for antibiotic treatment, I will offer a delayed/backup prescription first

Nurses	The next time, I see an antibiotic prescription which has continued beyond seven days without specified duration, I will highlight this to the doctors<br>The next time I am administering antibiotics, I will prompt the prescriber to review and document a decision 48 hours after the patient has started on antibiotics<br>The next time, I am giving a patients antibiotics (on discharge from hospital or via PGD), I will inform the patient on the indication for the antibiotics and that any left-over should be returned to a pharmacy, not shared or reused<br>As a nurse prescriber, the next time I want to prescribe an antibiotic for a self-limiting infection for a patient with high expectations for antibiotic treatment, I will consider delayed/backup prescription<br>As a nurse prescriber, the next time I decide not to prescribe a patient with an antibiotic for a self-limiting infection, I will give them the TARGET antibiotics patient information leaflet to support their self-care
Other Healthcare Professionals	I will encourage clients/patients and colleagues to become Antibiotic Guardians<br>In the week of EAAD (18th November) I will take the antibiotic quiz on the EAAD resources<br>I will champion promotional activity for EAAD within my organisation or local area
Pharmacy Teams	I will check that antibiotic prescriptions comply with local guidance and query those that do not<br>When handing out a prescription that includes antibiotics, I will provide the following key messages/ask the following questions • You have been prescribed antibiotics for xxxx infection (ask if you do not have the information available eg in community pharmacy) • Check if there are any known allergies • Take as prescribed (state dose, frequency and duration) • Do not share your antibiotics with other or reuse them after the stated duration … • Extra self-care information for infection management • Check they have had the flu vaccine (when applicable)<br>I will undertake one of the recommended CPD activities or e-challenges from the EAAD resources page during EAAD week<br>The next time a customer presents with a self-limiting infection of coughs/colds I will use the patient information leaflet to explain the potential duration of illness and how to treat their symptoms
Primary Care Prescribers	When I see a patient with a self-limiting illness, I will discuss methods of controlling symptoms rather than prescribing antibiotics<br>I will ensure all prescribers in my practice including locums have easy access to the local antibiotic guidance<br>When I see a child with a respiratory tract infection (coughs, colds, sore throats, and ear aches) I will use the TARGET - Guide to treat your infection - booklet rather than prescribe antibiotics. Available at http://www.rcgp.org.uk/clinical-and-research/target-antibiotics-toolkit.aspx

	The next time I intend to prescribe antibiotics for a self-limiting infection to a patient with high expectations of antibiotic treatment, I will use a delayed/backup prescription<br><br>I will undertake one of the recommended CPD activities on the EAAD resources page during EAAD week
Secondary Care Prescribers	If I prescribe an antibiotic then I will document indication and duration on the drug chart in line with Start Smart then Focus AMS guidance<br><br>I will undertake one of the recommended CPD activities on the EAAD resources page during EAAD week
Veterinary Teams	If there is a need to prescribe antibiotics I will use narrow spectrum drugs wherever possible<br><br>When dispensing antibiotics I will explain the importance of following the labelling instructions and giving the full course<br><br>I will ensure that there are effective cleaning and disinfection protocols in place to minimise the spread of bacteria between patients within the veterinary practice premises<br><br>If a treatment does not appear to work, I will advise a different course of action and report the treatment failure to the Veterinary Medicines Directorate (VMD)<br><br>I will display posters and reading material in my waiting room to help public understanding on antimicrobial resistance
Adults	For infections that our bodies are good at fighting off on their own, like coughs, colds, sore throats and flu, I pledge to try treating the symptoms for five days rather than going to the GP<br><br>It is vital we prevent antibiotics from getting into the environment. I pledge to always take any unused antibiotics to my pharmacy for safe disposal<br><br>For infections that our bodies are good at fighting off on their own, like coughs colds sore throats and flu, I pledge to talk to my pharmacist about how to treat the symptoms first rather than going to the GP
Families	For illness that our bodies are good at fighting off on their own, like coughs, colds, sore throats and flu, I pledge to talk to my pharmacist about how to treat my child’s symptoms first rather than going to the GP<br><br>Washing your hands properly is the single best way to prevent the spread of infections. My family pledges to help cut the need for antibiotics by always washing our hands with soap and water for about 30 seconds (about the same time it takes to sing A, B, C, D song)<br><br>I will visit the ebug website (www.e-bug.eu) with my child(ren) and take one of the antibiotic awareness quizzes together
Farmers	To help reduce the need for antibiotics I will evaluate my biosecurity and husbandry measures

	If my vet prescribes antibiotics I will give them in accordance with the instructions on the label and make sure that the full course is given and that any surplus is returned or disposed of correctly<br><br>If I have a disease on my farm I will work with my vet to identify preventative measures and help diagnose the cause in order to inform accurate prescribing and stop spread or re-infection
Pet Owners	To help reduce the need for antibiotics I will keep my animal healthy through exercise, good nutrition, relevant vaccination, and by having regular veterinary health checks<br><br>If my pet(s) are prescribed antibiotics by my vet, I will use them as instructed on the label and not give them to another animal<br><br>If my vet prescribes antibiotics I will give them in accordance with the instructions on the label and make sure that the full course is given
Students	I will practice and promote good hand hygiene at all times to reduce transmission of infection and resistance; the WHO FIVE moments for hand-hygiene is recommended http://bit.ly/hand-hygiene-WHO<br><br>For infections that our bodies are good at fighting off on their own, like coughs, colds, sore throats and flu, I pledge to talk to my pharmacist about how to treat the symptoms first rather than going to the GP<br><br>The next time I see an antibiotic prescribed, I will ask the prescriber about the indication and duration, to understand if this is in accordance to local and national guidelines<br><br>The next time I see that a recommended infection prevention practice is not being adhered to (e.g. hand washing), I will respectfully challenge my peers and healthcare workers<br><br>The next time I have an opportunity to carry out an audit, I will do one focused on antibiotic use or resistance<br><br>For my next clinical case study, I will include one focused on a common infection and how it should be treated in line with local and national guidelines
