# Supplementary material for: Impact of a United Kingdom-wide campaign to tackle antimicrobial resistance on self-reported knowledge and behaviour change
Source: BMC Public Health. 2016 May 12;16:393. doi: 10.1186/s12889-016-3057-2 (PMC4866421; doi:10.1186/s12889-016-3057-2)
Supplement: Additional file 2: — Online questionnaire used for the evaluation of the Antibiotic Guardian Campaign. (PDF 423 kb) [file 12889_2016_3057_MOESM2_ESM.pdf]

Evaluation of the Antibiotic Guardian Campaign 2014

Welcome

Thank you for choosing a pledge on www.antibioticguardian.com and becoming an Antibiotic Guardian. There are now over 12,500 Antibiotic Guardians.

We are inviting Antibiotic Guardians to complete this survey. Your answers will help us understand better what people think about the campaign and antibiotics and will help us improve our understanding in helping make it easier for you to meet your pledges.

Antibiotic Guardian is a year-round campaign; your feedback will help us improve the campaign. The publication of the evaluation will be shared with the Antibiotic Guardians.

We would be most grateful if you would complete the questionnaire in one sitting. It takes approximately 10 min to be completed. The information that we collect will be treated with the strictest confidence and is securely kept.

Thank you for taking the time to complete this questionnaire.

We would be grateful if you could complete the questionnaire by Tuesday 10th February.

European Antibiotic Awareness Day (EAAD) Planning Group
Antibiotic Guardian Campaign 2014
Public Health England

Antibiotic Guardian Pledge

1. Did you pledge as a:*
   - ○ Healthcare professional
   - ○ Member of the public

2. Specifically, did you pledge as a:
   Please tick one
   - ○ Primary Care Prescriber
   - ○ Secondary Care Prescriber
   - ○ Antimicrobial/Infection Prevention and Control Specialist
   - ○ Nurse
   - ○ Pharmacy Team
   - ○ Dentist
   - ○ Non-Medical Prescriber
   - ○ Other Healthcare Worker (e.g. Podiatrists, chiropodists, radiographers, therapists, social workers)
   - ○ Veterinary Practitioner
   - ○ Executive/Management/Government/Commissioner/Public Health
   - ○ Student

3. Specifically, did you pledge as:
   Please tick one
   - ○ Adult
   - ○ Family
   - ○ Pet owner
   - ○ Farmer
   - ○ Student

Your Antibiotic Guardian Pledge

4. Do you remember your specific pledge?*
   - Yes, I remember my pledge
   - Yes, but I had to check my confirmation email at the time of pledging
   - I remember the general pledge messages
   - Not really, I only remember a few details
   - I do not remember my pledge

5. Why did you choose your specific pledge?
   Please tick one
   - It was the pledge I thought I could successfully commit to
   - It was the one that I felt was most important
   - My friends/ family/professional colleagues chose the same pledge
   - No particular reason/cannot remember

6. What was the main reason you became an Antibiotic Guardian?
   Please tick one
   - I have personal experience with antibiotic resistance (e.g. I/I know someone who had a health problem related to antibiotic resistance)
   - I have professional experience with antibiotic resistance (e.g. I am a healthcare professional and I prescribe antibiotics to patients or I treat patients with antibiotic resistance)
   - A health professional explained to me the importance of antibiotic resistance and motivated me to be part of campaigns like the "Antibiotic Guardian"
   - My friends or family became "Antibiotic Guardians" and encouraged me to do the same
   - The materials used to promote the campaign (posters, leaflets, videos, social media, website) explained to me the importance of antibiotic resistance and motivated me to become an "Antibiotic Guardian"
   - It was mandatory for me (e.g. I work for an organisation where all the staff was asked to become an Antibiotic Guardian)
   - I know that antibiotic resistance is a very important problem for public health
   - I wanted to feel I am doing my part

7. Since you became an Antibiotic Guardian, have you acted in line with your pledge?*
   - Always
   - Most of the time
   - Some of the time
   - Occasionally
   - Never

8. You've indicated that you haven't always been able to act in line with your pledge. Please choose from the following as to why not.
   Please tick one
   - I have no control over it (e.g. I want to commit to my pledge but policies and procedures in my workplace makes this difficult)
   - I have had difficulties due to the expectations of others
   - I have not had the opportunity (e.g. If I pledged to not to use antibiotics the first 5 days of cold symptoms, I have not had the opportunity to act in accordance to my pledge because I have not had a cold since then)
   - I forgot about the pledge
   - I do not think the pledge is important

Antibiotics

9. Please indicate your agreement with the following statements regarding antibiotics.*
   Please tick one per row

	Strongly Agree	Agree	Tend to Agree	Tend to Disagree	Disagree	Strongly Disagree
Antibiotics do not work against colds and flu	○	○	○	○	○	○
Antibiotics should always been taken as prescribed	○	○	○	○	○	○
Antibiotics should never be saved for later or shared with others	○	○	○	○	○	○
Antibiotics are important medicines and should be preserved	○	○	○	○	○	○
Antibiotics are easy to get	○	○	○	○	○	○

Before becoming an Antibiotic Guardian

10. Before you heard about the Antibiotic Guardian campaign, were you aware of what antibiotic resistance is?

- ○ Yes
- ○ No

11. Before the campaign what were your thoughts on antibiotic resistance?
    Please tick all that apply
    Select at least 1 and no more than 6.

- ☐ I had no thoughts/feelings
- ☐ I knew it was a problem but was not sure exactly why
- ☐ I heard a lot about it but was confused
- ☐ I thought it was not an important issue
- ☐ I knew it was a problem and why
- ☐ I thought it was an important issue that the Government needs to do something about
- ☐ I thought it was an important issue that healthcare professionals need to do something about
- ☐ I thought it was an important issue that I can do something about

After becoming an Antibiotic Guardian

12. After becoming an Antibiotic Guardian, do you feel that you have acquired more knowledge on what antibiotic resistance is?

- ○ Yes
- ○ No

13. After the campaign what were your thoughts on antibiotic resistance?
Please tick all that apply
Select at least 1 and no more than 6.

- [ ] I had no thoughts/feelings
- [ ] I knew it was a problem but was not sure exactly why
- [ ] I heard a lot about it but was confused
- [ ] I thought it was not an important issue
- [ ] I knew it was a problem and why
- [ ] I thought it was an important issue that the Government needs to do something about
- [ ] I thought it was an important issue that healthcare professionals need to do something about
- [ ] I thought it was an important issue that I can do something about

14. Please indicate your agreement with the following statements regarding the Antibiotic Guardian campaign.*
Please tick one per row

	Strongly Agree	Agree	Tend to Agree	Tend to Disagree	Disagree	Strongly Disagree
I believe that the Antibiotic Guardian campaign will contribute to the prevention of antibiotic resistance	○	○	○	○	○	○
When I became an Antibiotic Guardian, I felt that I was part of a group of people that are trying to keep antibiotics active	○	○	○	○	○	○
I believe that the Antibiotic Guardian campaign has highlighted the importance of saving our antibiotics	○	○	○	○	○	○
Before I became an Antibiotic Guardian, I was acting in line with what I pledged	○	○	○	○	○	○
The Antibiotic Guardian campaign has changed the way I think about using antibiotics	○	○	○	○	○	○

15. Which of the sources listed below made the biggest impact on you (to become an Antibiotic Guardian)?
Please tick one

- ○ Print materials
- ○ YouTube video
- ○ Social Media (Facebook, Twitter, LinkedIn etc.)
- ○ Antibiotic Guardian website
- ○ Colleagues
- ○ Friends
- ○ A combination of these
- ○ None of the above

16. I found the information regarding antibiotic resistance provided by the Antibiotic Guardian Campaign **understandable**:*
Please indicate your agreement with the above statement regarding each different source listed below.

	Strongly Agree	Agree	Tend to Agree	Tend to Disagree	Disagree	Strongly Disagree	Not Seen
Posters	○	○	○	○	○	○	○
Leaflets	○	○	○	○	○	○	○
Quizzes/Crosswords	○	○	○	○	○	○	○
YouTube Video	○	○	○	○	○	○	○
Social Media	○	○	○	○	○	○	○
Antibiotic Guardian Website	○	○	○	○	○	○	○
Antibiotic Guardian pledge system	○	○	○	○	○	○	○

17. I **learnt something new** from the information regarding antibiotic resistance provided by the Antibiotic Guardian Campaign:*
Please indicate your agreement with the above statement regarding each different source listed below.

	Strongly Agree	Agree	Tend to Agree	Tend to Disagree	Disagree	Strongly Disagree	Not Seen
Posters	○	○	○	○	○	○	○
Leaflets	○	○	○	○	○	○	○
Quizzes/Crosswords	○	○	○	○	○	○	○
YouTube Video	○	○	○	○	○	○	○
Social Media	○	○	○	○	○	○	○
Antibiotic Guardian Website	○	○	○	○	○	○	○
Antibiotic Guardian pledge system	○	○	○	○	○	○	○

18. In which of these sources did you see Antibiotic guardian mentioned/promoted?
Please tick all that apply

- ☐ Facebook
- ☐ Twitter
- ☐ YouTube
- ☐ E-mail from professional body
- ☐ Someone's email signature
- ☐ Conference
- ☐ Email Senior member of organisation
- ☐ Media TV
- ☐ Media Newspaper
- ☐ Professional body website
- ☐ My organisation website
- ☐ None of the above

19. Please indicate your agreement with the following statements regarding the Antibiotic Guardian campaign.

	Strongly Agree	Agree	Tend to Agree	Tend to Disagree	Disagree	Strongly Disagree
I think campaign is well promoted	○	○	○	○	○	○

20. Did you share the information you learnt from the Antibiotic Guardian campaign with others?

- ○ I shared the information via social media
- ○ I emailed information to others
- ○ I gave others printed materials
- ○ I told others about what I learned
- ○ I did not share the information

Your details

21. What is your age?
Drop down with age bands

--None--

- --None--
- under 12
- 12-17 years old
- 18-24 years old
- 25-34 years old
- 35-44 years old
- 45-54 years old
- 55-64 years old
- 65-74 years old
- 75 years or older
- prefer not to say

22. Are you:

- Male
- Female
- Prefer not to say

23. Are you in a health profession?*

- Yes
- No

24. Which one of the following social media networks do you mainly use?
Please tick one

- Twitter
- Facebook
- LinkedIn
- Google+
- I do not use social media networks

25. Are you directly connected to the healthcare system e.g. do you work in a healthcare organisation or health related professional body?

- Yes
- No

If yes to Q23

26. What type of health professional are you?

--None--

- Doctor - GP
- Doctor - Hospital
- Doctor - Junior Doctor (FY1-2)
- Doctor - Registrar
- Doctor - Consultant
- Doctor - Clinical Microbiologist
- Nurse - Community
- Nurse - Primary Care
- Nurse - Secondary Care
- Pharmacist - Community
- Pharmacist - Primary Care
- Pharmacist - Secondary Care
- Pharmacy technician
- Allied healthcare professional (e.g. paramedic, podiatrist, radiographer, social worker)
- Clinical Veterinarian
- Veterinary Nurse
- Veterinary Practitioner
- Public Health Practitioner
- Student
- Other

27. Would you be interested in actively promoting the Antibiotic Guardian campaign to your friends, family and colleagues? (this could be as simple as sharing the website with them and asking for their feedback)

- Yes
- No

Thank you very much for completing the questionnaire, your feedback is highly valuable.

If you have additional comments or questions or feedback on how we can further promote the Antibiotic Guardian campaign, please do so in the comment box below.

Optional: Comments / Questions / Feedback.
(Free text paragraph box)
